# Whole-exome sequencing in 168 Korean patients with inherited retinal degeneration

**DOI:** 10.1186/s12920-021-00874-6

**Published:** 2021-03-10

**Authors:** Dae Joong Ma, Hyun-Seob Lee, Kwangsoo Kim, Seongmin Choi, Insoon Jang, Seo-Ho Cho, Chang Ki Yoon, Eun Kyoung Lee, Hyeong Gon Yu

**Affiliations:** 1 Retinal Degeneration Research Lab, Biomedical Research Institute, Seoul National University Hospital, Seoul, Republic of Korea; 2 Department of Ophthalmology, Hallym University Kangnam Sacred Heart Hospital, Seoul, Republic of Korea; 3 Genomics Core Facility, Translational Research Institute, Biomedical Research Institute, Seoul National University Hospital, Seoul, Republic of Korea; 4 Transdisciplinary Department of Medicine and Advanced Technology, Seoul National University Hospital, Seoul, Republic of Korea; 5 Biomedical Research Institute, Seoul National University Hospital, Seoul, Republic of Korea; 6 Department of Ophthalmology, College of Medicine, Seoul National University, Seoul, Republic of Korea

**Keywords:** Whole-exome sequencing, Inherited retinal degeneration, Retinitis pigmentosa

## Abstract

**Background:**

To date, no genetic analysis of inherited retinal disease (IRD) using whole-exome sequencing (WES) has been conducted in a large-scale Korean cohort. The aim of this study was to characterise the genetic profile of IRD patients in Korea using WES.

**Methods:**

We performed comprehensive molecular testing in 168 unrelated Korean IRD patients using WES. The potential pathogenicity of candidate variants was assessed using the American College of Medical Genetics and Genomics and the Association for Molecular Pathology variant interpretation guidelines, in silico prediction tools, published literature, and compatibility with known phenotypes or inheritance patterns.

**Results:**

Causative variants were detected in 86/168 (51.2%) IRD patients, including 58/107 (54.2%) with retinitis pigmentosa, 7/15 (46.7%) with cone and cone-rod dystrophy, 2/3 (66.6%) with Usher syndrome, 1/2 (50.0%) with congenital stationary night blindness, 2/2 (100.0%) with Leber congenital amaurosis, 1/1 (100.0%) with Bietti crystalline dystrophy, 1/1 (100.0%) with Joubert syndrome, 9/10 (90.0%) with Stargardt macular dystrophy, 1/10 (10.0%) with vitelliform macular dystrophy, 1/11 (9.1%) with other forms of macular dystrophy, and 3/4 (75.0%) with choroideraemia. *USH2A*, *ABCA4*, and *EYS* were the most common causative genes associated with IRD. For retinitis pigmentosa, variants of *USH2A* and *EYS* were the most common causative gene mutations.

**Conclusions:**

This study demonstrated the distribution of causative genetic mutations in Korean IRD patients. The data will serve as a reference for future genetic screening and development of treatment modalities for Korean IRD patients.

**Supplementary Information:**

The online version contains supplementary material available at 10.1186/s12920-021-00874-6.

## Background

Inherited retinal degeneration (IRD) is a group of clinically and genetically heterogeneous diseases, including retinitis pigmentosa (RP) and allied diseases, characterised by the progressive loss of photoreceptors and/or retinal pigment epithelial cells due to genetic anomalies related to the phototransduction cascade, retinal transcription factor-related pathway, RNA splicing machinery, retinal metabolism, retinal cell structure, and ciliary structure and function [1, 2]. To date, more than 270 genes have been identified for IRD, as listed in the Retinal Information Network [3].

Presently, a diagnosis of IRD is based mainly on clinical findings, including the primarily involved anatomical location or cell types, disease progression, and involvement of additional organs [1, 4]. However, the variable age of onset, genotypic heterogeneity (one phenotype can be caused by multiple genes), phenotypic heterogeneity (various mutations in a single gene result in numerous phenotypes), incomplete penetrance, unclear inheritance, and progressive nature of IRD, impede a definitive diagnosis [1]. Thus, molecular genetic testing is imperative for a definitive IRD diagnosis.

We previously reported that the hereditary features and mutation profile of Korean patients with IRD differed from those of Chinese and Japanese patients, as well as patients of other ethnicities [5–7]. However, no genetic analysis of IRD using whole-exome sequencing (WES) has been conducted in a large-scale Korean cohort. The aim of this study was to investigate the mutation spectrum and frequency in a large-scale Korean IRD cohort using WES.

## Methods

### Patients

A cohort of 168 unrelated Korean patients with IRD was recruited from the Department of Ophthalmology, Seoul National University Hospital (SNUH), from October 2008 to January 2019. A detailed family history was obtained from each patient to construct a pedigree. All patients underwent a detailed ophthalmic history and ophthalmic examinations, including best-corrected visual acuity, slit-lamp biomicroscopy, fundus photography, optical coherence tomography, fluorescein angiography, visual field tests, and full-field electroretinograms. Clinical diagnosis was determined by at least two retinal specialists (DJM and UCP).

### Comparison of exome capture products

In preparation for higher-throughput exome sequencing using the NextSeq500 (Illumina, San Diego, CA, USA), we evaluated the evenness, depth, duplication rate, and fraction sequenced to ≥ 20 × depth of three exome capture products: SureSelect Human All Exon v6 (Agilent Technologies, Santa Clara, CA, USA) with 150 bp paired-end reads or with 350 bp paired-end reads, and xGen Lockdown panel (Integrated DNA Technologies, Coralville, IA, USA) with 150 bp paired-end reads. The evenness score describes the uniformity of the base coverage over the target regions, and was calculated according to the method described by Mokry et al. [8]. In addition, sensitivity and positive predictive value of variant calling in comparison to a reference genome were calculated. To reduce experimental variables and minimise bias between libraries, all libraries were prepared using the Topomize DNA LT Library Prep Kit (MCLAB, South San Francisco, CA, USA). Agilent captures were hybridised as single sample reactions using 500 ng of library as input. Integrated DNA Technologies captures were hybridised as pools of three samples using 500 ng of library input. All hybridisation and post-hybridisation captures, and washes were performed according to each respective manufacturer’s protocol. The same samples were used for all exon capture methods (three sets of three identical samples).

### WES

WES was performed at the Genomics core facility of the Center for BioMarkers (SNUH, South Korea) using an Illumina NextSeq500. For sequencing on the NextSeq500 platform, libraries were generated using the Topomize DNA LT Library Prep Kit and the hybridisation capture of DNA libraries was performed with xGen Lockdown panels to generate 150 bp paired-end reads. Sequenced reads were aligned to UCSC hg19 human reference genome downloaded from the GATK website (https://gatk.broadinstitute.org). Alignment of the sequence reads, indexing of the reference genome, variant calling, and annotation were performed with a pipeline based on Burrows-Wheeler Alignment [9] using BaseSpace Onsite (Illumina). Variants were annotated using Alamut-HT and visualised on Alamut Viewer 2.2 (Interactive Biosoftware, Rouen, France).

### Bioinformatic analysis

After read mapping, the output alignment file was sorted using the Genome Analysis Tool Kit (GATK) AddOrReplaceReadGroups [10]. Potential polymerase chain reaction duplicates were removed using GATK MarkDuplicates. GATK BaseRecalibrator and ApplyBQSR were further used to recalibrate the base quality scores. After these pre-processing steps, germline variants were called using GATK HaplotypeCaller, while single nucleotide polymorphisms and insertion-deletion polymorphisms were annotated using Annotate Variation software [11]. After variant annotation, low-quality variants that had a read depth < 10, variant allele fraction < 10%, or variant read count < 2 were removed.

Variants were prioritised based on their presence amongst the 271 genes associated with IRD in RetNet [3]. Variants were classified according to the recent recommendations of the American College of Medical Genetics and Genomics and the Association for Molecular Pathology (ACMG/AMP) using InterVar, as follows: pathogenic, likely pathogenic, uncertain significance, likely benign, or benign variant [12–14].

Variants were considered pathogenic when one of the following criteria was met: (1) mutation was previously described as disease-causing in the Human Gene Mutation Database or published literature [15]; (2) mutation was classified as pathogenic or likely pathogenic according to the ACMG/AMP guidelines; (3) mutation was predicted as likely damaging, deleterious, disease-causing, and medium or high impact by more than half of the six pathogenicity prediction tools, including SIFT [16], PolyPhen‐2 HDIV and PolyPhen‐2 Hvar [17], MutationTaster [18], MutationAssesor [19], and FATHMM [20]; or (4) a resultant protein truncation mutation, such as a nonsense or frameshift (insertion or deletion) mutation, was identified. All variants not compatible with known phenotypes or inheritance patterns were excluded.

Patients were determined to have causative variant(s) when (1) one pathogenic variant was present in a gene with autosomal dominant (AD) or X-linked (XL) inheritance; (2) two heterozygous variants or one homozygous pathogenic variant was present in a gene with autosomal recessive (AR) inheritance. Patients were determined to have possible causative variant(s) when one heterozygous pathogenic variant was present in a gene with AR inheritance. Patients were determined to have no causative variants when no (possible) pathogenic variant was detected.

### Variant validation

Additional Sanger sequencing was performed for all pathogenic variants with a coverage < 20 reads. Only 5/177 variants (2.8%) have coverage < 20 reads, all of which were successfully sequenced. The concordance rate was 100.0% (5/5).

### Statistical analysis

To compare the performance of the exon capture products, we applied the Mann–Whitney U-test. All statistical analyses were performed using SPSS software for Windows, version 22.0 (IBM, Armonk, NY, USA). A value of *P* < 0.05 was regarded as statistically significant.

## Results

### Patients

We analysed samples from 168 unrelated Korean patients with IRD (77 females, 91 males). The mean patient age was 42.8 ± 15.9 years (range: 10.4–85.4; median: 37.1). Among these patients, 133 (79.2%) had photoreceptor disease, 33 (19.6%) had macular disease, and 2 (1.2%) had choroideraemia (Table 1). RP was the most common form of IRD (60.7%).

**Table 1:** Clinical details of 168 Korean patients with inherited retinal degeneration

Clinical details			n (%)
Clinical diagnosis	Photoreceptor disease	Retinitis pigmentosa	102 (60.4%)
		Cone and cone-rod dystrophy	22 (13.1%)
		Usher syndrome	3 (1.8%)
		Congenital stationary night blindness	2 (1.2%)
		Leber congenital amaurosis	2 (1.2%)
		Bardet–Biedl syndrome	1 (0.6%)
		Joubert syndrome	1 (0.6%)
	Macular disease	Stargardt macular dystrophy	13 (7.7%)
		Vitelliform macular dystrophy	11 (6.5%)
		North Carolina macular dystrophy	1 (0.6%)
		Other macular dystrophy	8 (4.8%)
	Choroideraemia		2 (1.2%)
Inheritance pattern	Autosomal recessive		67 (39.9%)
	Autosomal dominant		35 (20.8%)
	X-linked		6 (3.6%)
	Simplex		60 (35.7%)

Based on their family pedigrees, 67 (39.9%) of the patients were presumed to have IRD with AR inheritance, 35 (20.8%) with AD inheritance, and 6 (3.6%) with XL inheritance. Sixty (35.7%) patients were the only affected individuals in their family (simplex cases).

### Comparison of exome capture products

The evenness score and sensitivity were significantly higher for the SureSelect Human All Exon v6 with 350 bp paired-end reads than for the other capture products (Table 2). The SureSelect Human All Exon v6 with 150 bp paired-end reads and xGen Lockdown panel produced statistically equivalent results. The depth and duplication rate were highest for the SureSelect Human All Exon v6 with 150 bp paired-end reads, then for the xGen Lockdown panel, and lowest for the SureSelect Human All Exon v6 with 350 bp paired-end reads. The positive predictive value was highest for the xGen Lockdown panel, then for the SureSelect Human All Exon v6 with 150 bp paired-end reads, and lowest for the SureSelect Human All Exon v6 with 350 bp paired-end reads.

**Table 2:** Comparison of exome capture products

	SureSelect Human All Exon v6		xGen Lockdown panel (n = 9^a^)	*P *value^b^	*P* value^c^	*P* value^d^
	150-bp paired-end reads (n = 9^a^)	350-bp paired-end reads (n = 9^a^)				
	Mean ± SD	Mean ± SD	Mean ± SD			
Evenness (%)	75.3 ± 5.7	79.9 ± 0.4	76.6 ± 0.1	< 0.001	0.258	< 0.001
Depth (x)	61.3 ± 3.8	47.6 ± 0.5	56.3 ± 1.1	< 0.001	< 0.001	< 0.001
Duplication rate (%)	33.4 ± 2.4	17.5 ± 0.2	22.9 ± 0.4	< 0.001	< 0.001	< 0.001
≥ 20 × depth fraction (%)	87.9 ± 7.4	90.8 ± 0.6	91.3 ± 0.2	0.258	0.258	0.258
Sensitivity (%)	94.9 ± 4.0	97.8 ± 0.1	96.5 ± 0.2	< 0.001	0.258	< 0.001
Positive predictive value (%)	74.9 ± 0.7	73.8 ± 0.2	76.1 ± 0.2	< 0.001	< 0.001	< 0.001

*SD* standard deviation

^a^Three sets of three identical samples

^b^*P *value comparing SureSelect Human All Exon v6 150-bp paired-end reads with 350-bp paired-end reads

^c^*P* value comparing SureSelect Human All Exon v6 150-bp paired-end reads with xGen Lockdown panel

^d^*P *value comparing SureSelect Human All Exon v6 350-bp paired-end reads with xGen Lockdown panel

The fraction of nucleotides covered to a depth ≥ 20 × did not differ among the exome capture products. In summary, the xGen Lockdown panel demonstrated noninferiority to the SureSelect Human All Exon v6, and further exome capture for WES was performed using the xGen Lockdown panel.

### Identification of pathogenic variants

**Fig. 1:**
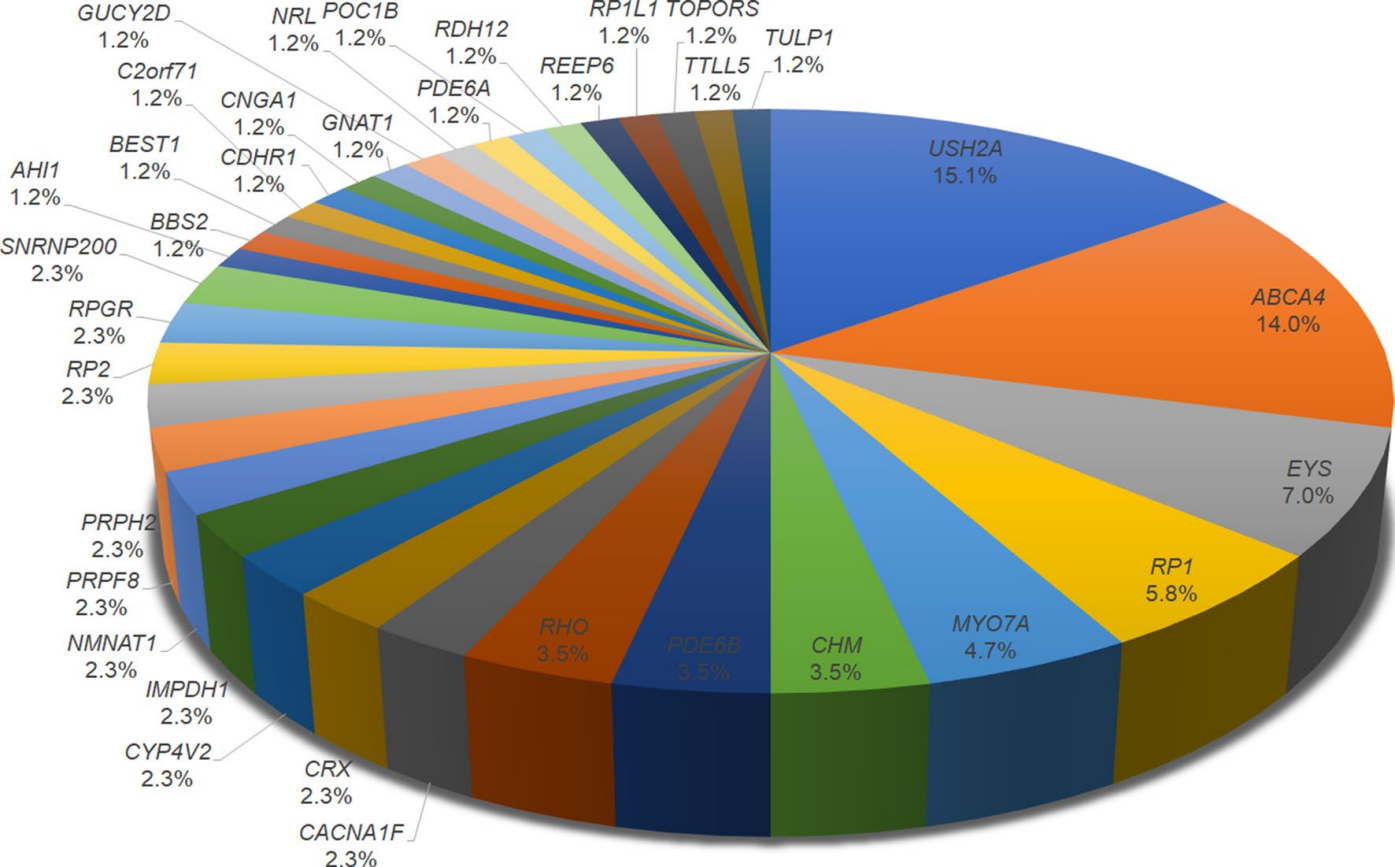
*USH2A* and *ABCA4* mutations were the most common causative variants among inherited retinal degeneration patients. All data are from the present study

We identified disease-causing variants in 86 (51.2%) patients (Additional file 1: Table S1). In all, 147 mutant alleles were identified in 35 known IRD-related genes. Among these mutant alleles, 96 (65.3%) had a previously reported association with IRD, and 51 (34.7%) were novel variants in defined IRD genes. Amongst the identified disease-associated variants, 89 (60.5%) were missense variants, 26 (17.7%) were nonsense variants, 25 (17.0%) were frameshift deletions or insertions, and 7 (4.8%) were predicted to affect splicing. *USH2A* was the most common causative gene amongst the known disease-associated genes (15.1% of cases), followed by *ABCA4* (14.0%) (Fig. 1).

Table S1 (see Additional file 1) [21–62]

### Molecular diagnosis redefined inheritance pattern and clinical diagnosis

**Fig. 2:**
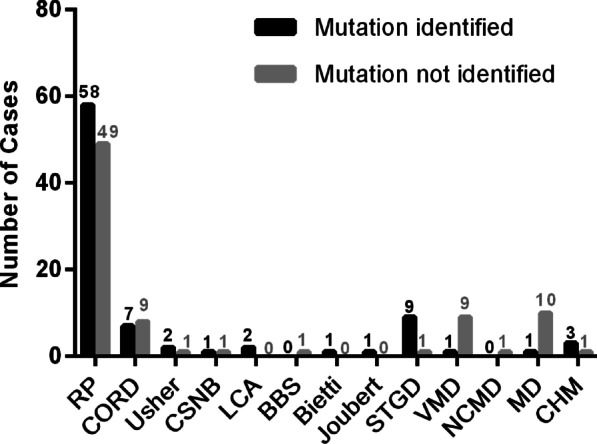
The number of detected pathogenic mutations for each disease phenotype. *BBS* Bardet–Biedl syndrome, *Bietti* Bietti crystalline dystrophy, *CHM* choroideraemia, *CSNB* congenital stationary night blindness, *CORD* cone and cone-rod dystrophy, *Joubert* Joubert syndrome, *LCA* Leber congenital amaurosis, *MD* other macular dystrophy, *NCMD* North Carolina macular dystrophy, *RP* retinitis pigmentosa, *STGD* Stargardt macular dystrophy, *Ushe*r Usher syndrome, *VMD* vitelliform macular dystrophy

All patients who received a definite molecular diagnosis had their clinical diagnosis and inheritance pattern redefined based on the genetic findings (Additional file 1: Table S1). Figure 2 shows a comparison of the mutation detection rates for various IRDs after reclassification. The following are two representative cases.

**Fig. 3:**
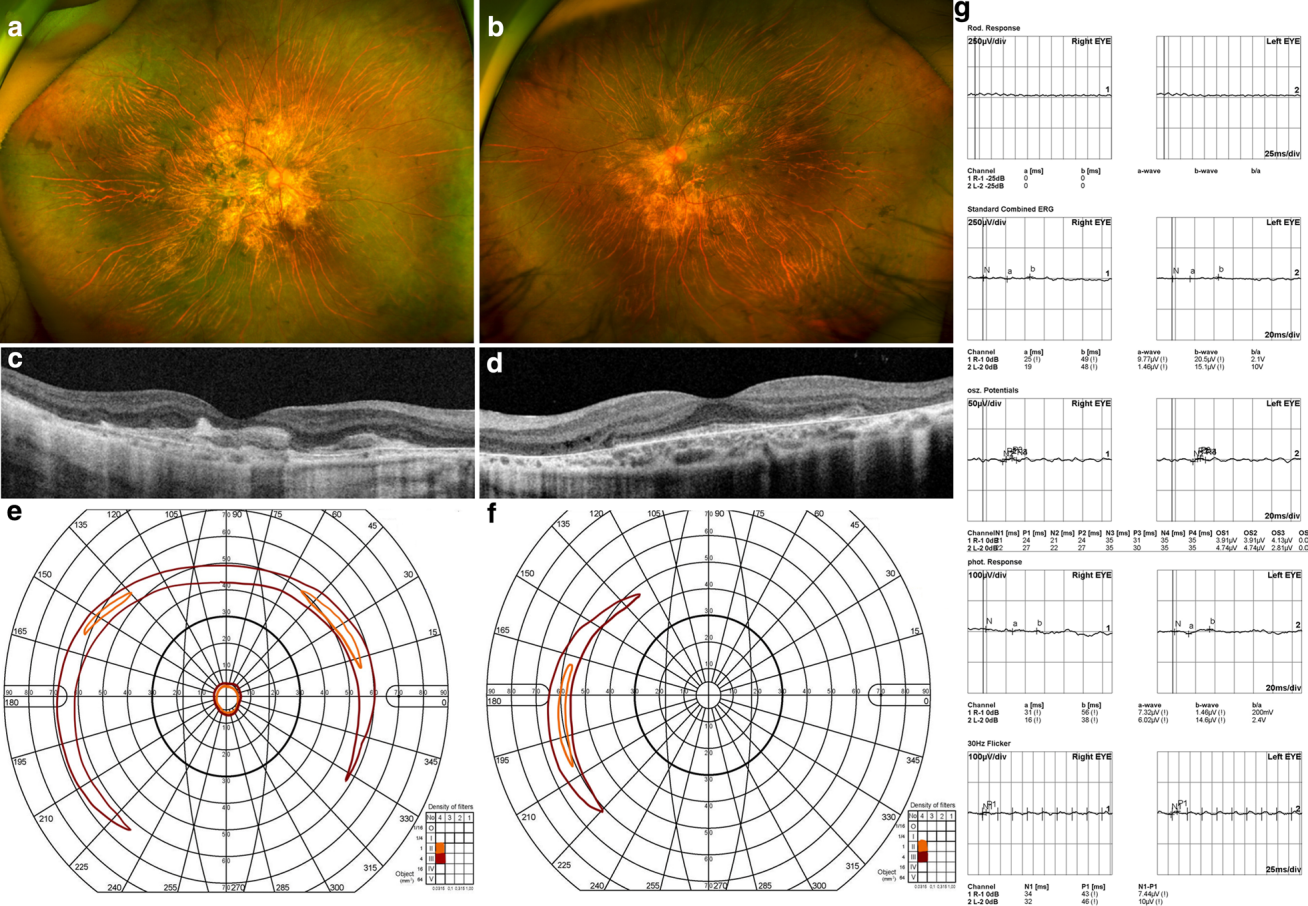
Clinical phenotypes of case 55, who carries a hemizygous mutation in the *CHM* gene. **a**, **b** Colour fundus photograph. **c**, **d** Optical coherence tomography images. **e**, **f** Vision field diagram. **g** Electroretinography recording

**Fig. 4:**
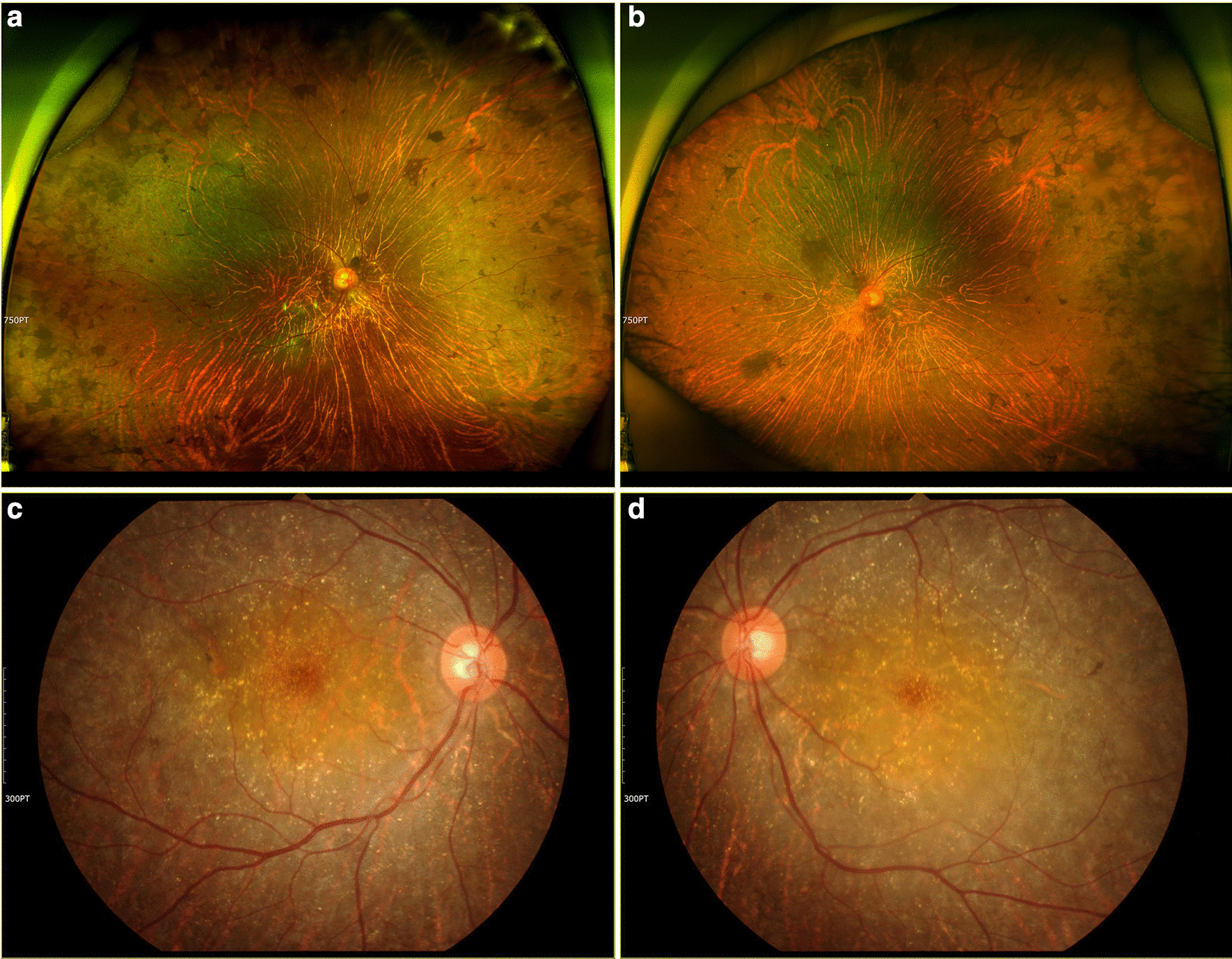
Clinical phenotypes of case 147, who carries compound heterozygous mutations in the *CYP4V2* gene. **a**, **b** Colour fundus photographs taken at the time of genetic analysis. **c**, **d** Colour fundus photographs from 9 years prior to the study

Case 55, who had been clinically diagnosed with RP, had macular-sparing photoreceptor degeneration with diffuse bone spicule pigmentation and degeneration of the retinal pigment epithelium and choriocapillaris (Fig. 3). WES identified causative variants in the *CHM* gene, and the clinical diagnosis for case 55 was redefined as choroideraemia. Case 147 was clinically diagnosed with RP with generalised photoreceptor, retinal pigment epithelium, and choriocapillaris degeneration with diffuse bone spicule pigmentation (Fig. 4). WES identified causative variants in the *CYP4V2* gene, and the clinical diagnosis for case 147 was redefined as Bietti crystalline dystrophy. Fundus photographs from 9 years prior to sequencing showed many shiny yellow deposits in the posterior pole, which are consistent with Bietti crystalline dystrophy.

Fifty-five patients (32.7%) had their inheritance patterns redefined based on the genetic findings. Of the 37 simplex cases, 35 patients (94.6%) were reclassified as having AR inheritance, while AD inheritance and XL inheritance were diagnosed in one patient each (2.7%).

#### Non-syndromic RP

**Fig. 5:**
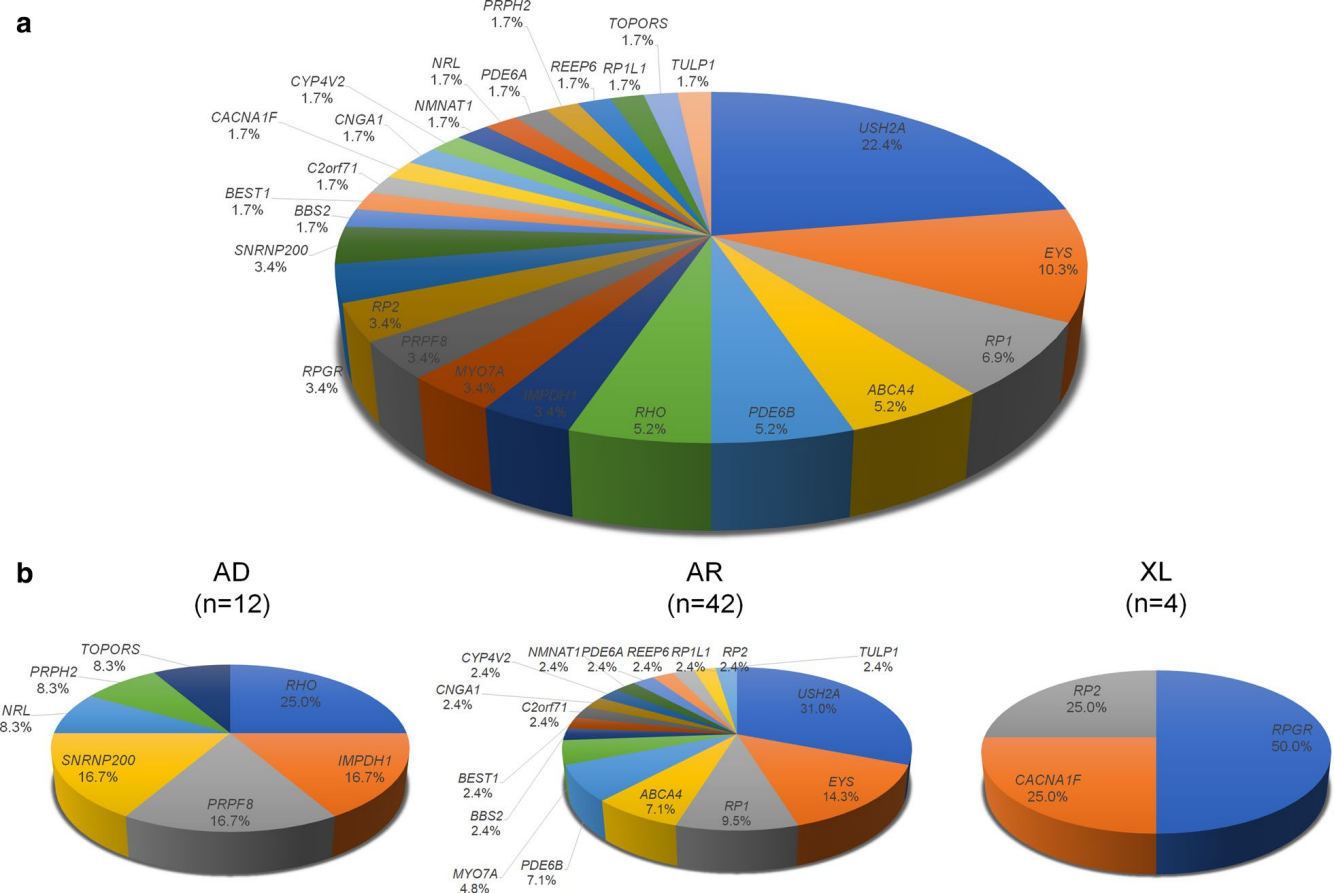
Percentages of causative genes identified in 58 retinitis pigmentosa patients. Mutations in *EYS* and *USH2A* were the most common in the population (**a**). The proportion of causative genes based on inheritance pattern is shown in **b**. All data are from the present study

After refinement of clinical diagnosis, 107 patients received molecular diagnosis of RP, comprising 63.7% of the total cohort. We identified causative variants for 58 of the 107 (54.2%) patients, including 96 variants in 26 genes (Additional file 1: Table S1). Variants in *USH2A* were the most common, which were carried by 13 patients (22.4%), followed by *EYS* variants in six patients (10.3%; Fig. 5).

*RHO* (3, 25.0%) was the most commonly identified mutated gene in 12 RP patients with AD inheritance (adRP; 20.7%), *USHA2A* (13, 31.0%) and *EYS* (6, 14.3%) were the most commonly identified mutated genes in 42 RP patients with AR inheritance (arRP; 72.4%), and *RPGR* (2, 50%) was the most commonly identified mutated gene in four RP patients with XL inheritance (xlRP, 6.9%).

#### Cone and cone-rod dystrophy

Amongst the 15 cone and cone-rod dystrophy (CORD) patient cases, we identified causative variants in seven patients (46.7%) (Additional file 1: Table S1). *CRX* (2, 28.6%) was the most commonly identified mutated gene.

#### Stargardt macular dystrophy

Of the 13 patients with an initial clinical diagnosis of Stargardt macular dystrophy (STGD), three patients were reclassified as having CORD or other macular dystrophy based on the genetic findings. WES revealed causative variants for 9 out of the 10 remaining STGD patients and a possible causative variant for the tenth patient. All identified causative variants were of the *ABCA4* gene (Additional file 1: Table S1).

### Possible pathogenic variants were identified

A further 17.9% (30/168) of the patients received a possible molecular diagnosis based on identification of a single pathogenic variant in a gene known to be associated with AR IRD that was compatible with known phenotypes or inheritance patterns (Additional file 2: Table S2). Overall, 69.0% (116/168) of study participants received a definite or possible molecular diagnosis after WES testing.

Table S2 (see Additional file 2) [21–70]

## Discussion

In this study, we employed a single genetic test, WES, for the genetic diagnosis of 168 unrelated Korean patients with different types of IRD. Using this single test, we molecularly diagnosed 86 (51.2%) patients. To the best of our knowledge, this is the first study to evaluate clinical diagnostic accuracy and causative genes in Korean patients with various IRD using a WES approach.

Targeted sequence capture is used to isolate and enrich specific genomic regions prior to massively parallel sequencing. Although the cost of WES is gradually decreasing, it remains expensive for clinical use. Herein, we used pre-capture pooling with targeted sequence capture to reduce the reagent cost and hands-on time. As pre-capture pooling using xGen Lockdown panel was not inferior to routine post-capture pooling using SureSelect Human All Exon v6, we successfully utilised the xGen panel for WES in Korean IRD patients. We identified 147 causative variants in 35 known IRD-related genes, including 51 (34.7%) novel variants. However, most of these novel variants were potentially causative which were annotated by computational tools. Functional validation would be required to derive a definite causality of these potentially causative variant.

Several studies have reported that the hereditary features and causative genes of IRD vary among ethnicities, even in geopolitically close Asian countries [5–7, 29, 71–73]. However, only a few reports have been published on the mutation spectrum of a large-scale Korean cohort with IRD. Our previous study using targeted exome sequencing (TES) of 53 RP-related genes in 62 Korean patients with non-syndromic RP revealed causal variants in 50.0% of the patients [7]. *PRPF31* mutations (17.6%) were the most frequently found causative variants, followed by mutations in *EYS*, *PDE6B*, *RHO*, *RP1*, and *RP2* (11.8% each). A recent study reported the TES results for 204 IRD-related genes in 86 Korean patients with IRD [74], as well as the molecular diagnoses rate for 44.2% of the patients. In RP, *EYS* mutations (22.2%) were the most frequent causative variants, followed by mutations in *PED6B* (16.7%), *PED6A* (11.1%), and *USH2A* (11.1%). In the present study, *USH2A* mutations (22.4%) were the most common causative variants in RP, followed by variants in *EYS* (10.3%), *RP1* (6.9%), and *ABCA4*, *PED6B*, and *RHO* (5.2% each) mutations. The differences in the mutation spectra amongst the Korean studies appears to largely originate from selection of the sample population.

Moreover, the sample sizes of the two previous studies were much smaller than that of the present study making them more vulnerable to sampling error. In addition, the inclusion of many subjects from a single family can result in increased representation of a specific genetic mutation, thereby skewing the mutational spectrum. In the present study, we only included one proband from each family to maximise the representativeness of the cohort for the total population and to reduce sampling error. In addition, differing inheritance pattern distributions in the sampled populations may result in different distributions of causative mutations. Our previous study included a large proportion of subjects with AD inheritance, which resulted in a higher proportion of mutations in *PRPF31*, an AD inheritance gene [7]. In the present study, the distribution of the inheritance pattern for RP resembles that in a previous report on a large cohort of Korean RP patients [6].

The mutation spectrum of the patients in the present study resembles those established in recent large-scale studies in other Asian countries [21, 75]. For instance, the *USH2A* mutation was the most common in the present study, as in the Chinese study, and it was the second most common in the Japanese study. The second most common mutation was in the *EYS* gene, which was the most common in the Japanese study and the third most common in the Chinese study. These findings were similar to those of previous studies demonstrating ethnic differences between Asian and Caucasian populations, with a higher incidence of *EYS* mutations in arRP patients of an Asian background than Caucasian background [76]. The most common *EYS* mutation in our cohort was c.4957dupA; it was also common in Japanese RP patients, however, was rare, or not detected in European RP patients and Chinese RP patients [66, 77]. Meanwhile, the c.C8805A and c.C7394G *EYS* mutations, which were frequently observed in the Japanese RP cohort, were not detected in Korean studies [7, 74, 78]. This suggests that differences in the mutational spectra of IRD patients exist among East Asian countries, despite their geographical proximity, and emphasises the importance of obtaining reference data for individual nations or regions to determine the local genomic IRD landscape.

The prevalence of the *RP1* mutation was 6.9% in arRP cases in this study, which was higher than those among Japanese and Chinese arRP patients (1.7–2%) [29, 73]. Mutations in the *RP1* gene cause both AR and AD forms of RP, accounting for 5.5% of adRP and less than 1% of arRP [76]. Different explanations for the dominant/recessive mutation effect of the *RP1* gene have been proposed, however, the precise mechanism remains unclear. In addition, a recent study suggested that the phenotypic spectrum associated with *RP1* mutations should be expanded to CORD and macular dystrophy [79, 80]. In our cohort, Case 103, who showed well-demarcated macular atrophy with normal electroretinography findings (see Additional file 3) carried compound heterozygous nonsense (c.C5797T) and frameshift (c.649delG) mutations, and received a molecular diagnosis of *RP1*-associated AR macular dystrophy.

WES has clear advantages over TES for the molecular diagnosis of IRD. The heterogeneity of the genotype and phenotype, as well as the unclear inheritance patterns of IRD, make it difficult to select target genes for TES. In addition, more than 270 causative genes have been identified for IRD to date, and new causative genes continue to be discovered. To keep pace with the literature, researchers must redesign panels to incorporate new genomic regions at additional expense. In contrast, WES provides the advantage of re-evaluating previously analysed datasets when a novel gene associated with IRD is reported. In a study comparing WES and three commercial gene panels, WES discovered causative gene mutations in 42% of cases, which were not included in at least one commercial panel [81]. In the present study, six genes (*CYP4V2*, *NMNAT1*, *RP1L1*, *CACNA1F, BBS2*, and *REEP6*) out of 35 causative genes detected in our cohort were not included in at least one of the TES studies in Korean IRD patients [7, 74].

Among patients with macular disease, patients with STGD had the highest detection rate for causative variants (90.0%), however, patients with other forms had a poorer detection rate (9.5%). This is likely due to the presence of pathognomonic findings for the accurate clinical diagnosis of STGD. Initially, 13 patients with clinical diagnosis of STGD were included in this study. Three patients who were redefined as having another disease based on their molecular diagnosis did not have dark choroid rings on fluorescein angiography. In contrast, the remaining ten patients had dark choroid signs and a (probable) molecular diagnosis of STGD. This pathognomonic finding, the dark choroid sign, can differentiate STGD from other similar conditions including non-hereditary diseases [82]. Meanwhile, all vitelliform macular dystrophy cases in our cohort were clinically diagnosed as adult-onset vitelliform macular dystrophy with clinical findings of submacular vitelliform material and normal electrooculogram. The mean age of the vitelliform macular dystrophy patients was 64.5 years. These clinical features are similar to those of exudative age-related macular degeneration, choroidal neovascularisation, or central serous chorioretinopathy, which do not belong to the category of IRD. There is a possibility that some of our vitelliform macular dystrophy cases may arise from non-genetic conditions. This finding suggests that accurate clinical diagnosis can increase the efficiency of molecular diagnosis.

The 17.9% (30/168) of patients who received a possible molecular diagnosis may carry a second pathogenic variant in the same gene, which occurs in no- or low-coverage genetic regions [73]. In the exome sequencing approach, some genetic regions have low or no coverage. For example, repetitive regions, GC-rich regions, and regions with high homology are difficult to enrich. In addition, small copy number variations and deep-intronic regions cannot be detected via exome sequencing [83, 84]. In these patients, further analysis, such as direct sequence analysis or whole genome sequencing, is required.

In this study, we were unable to identify any causative variants in 31.0% (52/168) of the cases. Several explanations may account for these. The first explanation is the limitation of WES. Approximately 85% of known causative mutations occur in exonic regions that encode proteins, indicating that WES is unable to discover the cause of the remaining ~ 15% of causative mutations. In addition, some genetic regions have low or no coverage in the exome sequencing approach, as described above. The second explanation is potentially inaccurate clinical diagnosis. It is possible that some of our cohort have non-hereditary retinal conditions that phenotypically resemble IRD. Therefore, further study via direct sequence analysis or whole-genome sequencing with detailed clinical diagnosis is required to achieve an optimal detection rate.

## Conclusion

The present study screened the largest sample of Korean IRD patients to date and described the genetic characteristics of the cohort. Our data will serve as a basis for genetic counselling of Korean IRD patients and lay the groundwork for the upcoming era of gene therapy.

## Supplementary Information

**Additional file 1.** **Table S1**: Causative variants identified in 86 of 168 Korean inherited retinal degeneration probands.

**Additional file 2.** **Table S2**: Possible causative variants in 30 of 168 Korean inherited retinal degeneration probands.

**Additional file 3.** **Figure S1**: Phenotypes of case 103 who carries compound heterozygous mutations in the *RP1* gene.

## Data Availability

The datasets generated and/or analysed during the current study are available in the NCBI Sequence Read Archive (SRA) repository, Accession Number PRJNA690657.

## Abbreviations

IRD: Inherited retinal disease
WES: Whole-exome sequencing
RP: Retinitis pigmentosa
SNUH: Seoul National University Hospital
GATK: Genome Analysis Tool Kit
ACMG/AMP: American College of Medical Genetics and Genomics and the Association for Molecular Pathology
AR: Autosomal recessive
AD: Autosomal dominant
XL: X-linked
CORD: Cone and cone-rod dystrophy
Usher: Usher syndrome
LCA: Leber congenital amaurosis
BBS: Bardet–Biedl syndrome
Bietti: Bietti crystalline dystrophy
CSNB: Congenital stationary night blindness
Joubert: Joubert syndrome
VMD: Vitelliform macular dystrophy
STGD: Stargardt macular dystrophy
NCMD: North Carolina macular dystrophy
MD: Other macular dystrophy
CHM: Choroideraemia
adRP: RP with AD inheritance
arRP: RP with AR inheritance
xlRP: RP with XL inheritance
TES: Targeted exome sequencing
