# Supplementary material for: Whole-exome sequencing in 168 Korean patients with inherited retinal degeneration
Source: BMC Med Genomics. 2021 Mar 10;14:74. doi: 10.1186/s12920-021-00874-6 (PMC7945660; doi:10.1186/s12920-021-00874-6)
Supplement: Supplementary file 3 — Additional file 3. Figure S1: Phenotypes of case 103 who carries compound heterozygous mutations in the RP1 gene. [file 12920_2021_874_MOESM3_ESM.docx]

**Figure S1.** Phenotypes of case 103 who carries compound heterozygous mutations in the *RP1* gene.


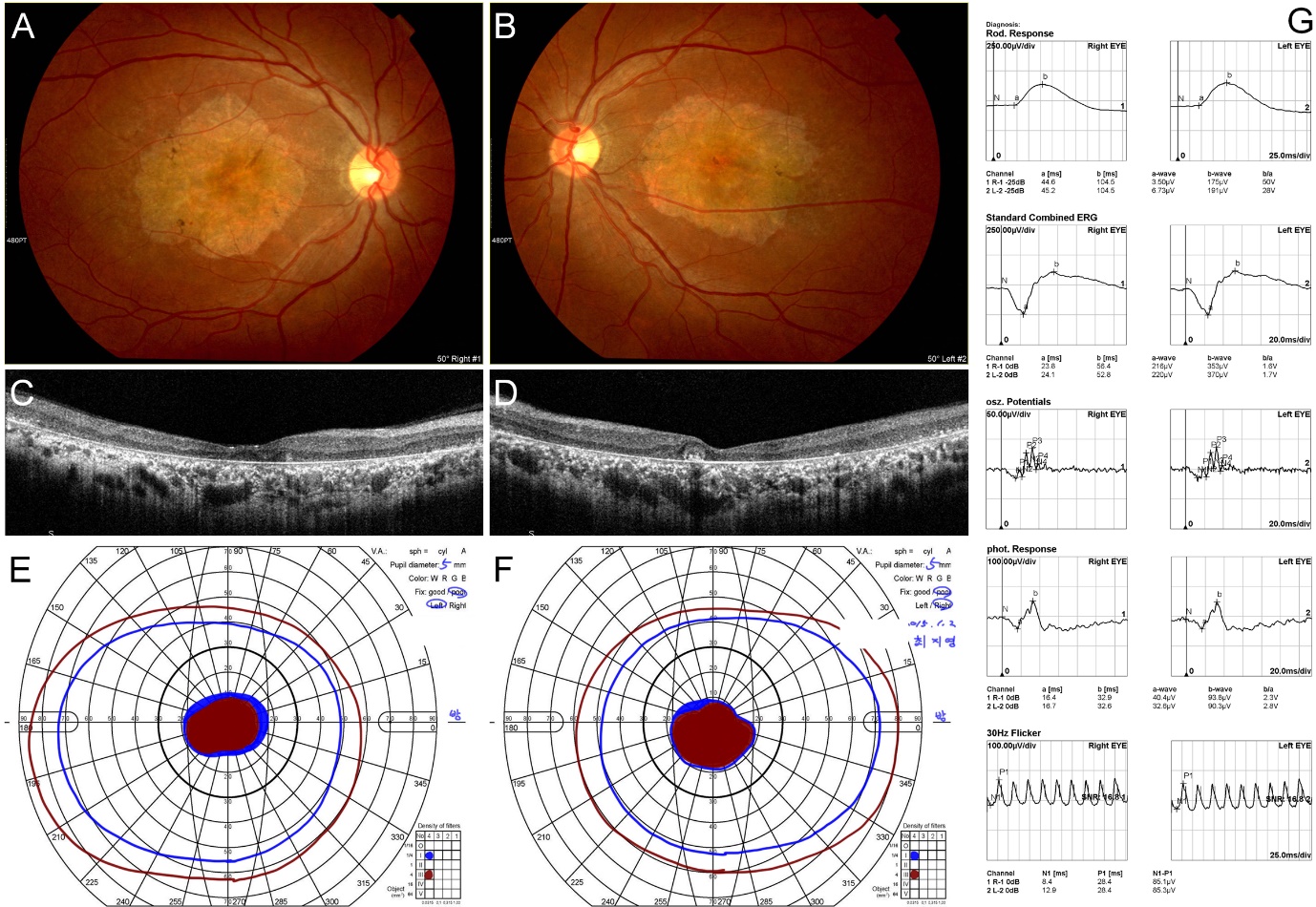


(A,B) Color fundus photograph. (C,D) Optical coherence tomography images. (E,F) Vision field diagram. (G) ERG recording.
